# Fibrinogen-to-prealbumin and C-reactive protein-to-prealbumin ratios as prognostic indicators in severe fever with thrombocytopenia syndrome

**DOI:** 10.3389/fcimb.2024.1397789

**Published:** 2024-06-10

**Authors:** Fan Zhang, Xiao-Yi Liu, Jin-Ping Qiao, Wen-Tao He

**Affiliations:** ^1^ Department of Clinical Laboratory, The First Affiliated Hospital of Anhui Medical University, Hefei, Anhui, China; ^2^ Department of Clinical Nutrition, The First Affiliated Hospital of USTC, Division of Life Sciences and Medicine, University of Science and Technology of China, Hefei, Anhui, China

**Keywords:** C-reactive protein-to-prealbumin ratio, fibrinogen-to-prealbumin ratio, prognosis, risk factor, severe fever with thrombocytopenia syndrome

## Abstract

**Background:**

The primary aim of this study is to investigate the correlation between serum levels of fibrinogen-to-prealbumin ratio (FPR) and C-reactive protein-to-prealbumin ratio (CPR) and prognostic outcomes among patients with severe fever with thrombocytopenia syndrome (SFTS). SFTS, characterized by elevated mortality rates, represents a substantial public health challenge as an emerging infectious disease.

**Methods:**

The study included 159 patients with SFTS. Clinical and laboratory data were compared between the survival and death groups. Univariate and multivariate logistic regression analysis were utilized to identify independent risk factors for mortality. The predictive efficacy of FPR and CPR was evaluated using receiver operating characteristic (ROC) curve. Survival analysis was conducted using the Kaplan–Meier curve and the log-rank test was employed for comparison.

**Results:**

The death group exhibited significantly elevated levels of FPR and CPR compared to the survival group (*P* < 0.05). Multivariate logistic regression analysis confirmed that both FPR and CPR independently correlated with a poorer prognosis among patients with SFTS. The ROC curve analysis indicated that FPR and CPR had superior predictive capabilities compared to C-reactive protein and fibrinogen. Kaplan–Meier survival analysis demonstrated that patients with SFTS who have FPR > 0.045 (log-rank test; χ2 = 17.370, *P* < 0.001) or CPR > 0.05 (log-rank test; χ2 = 19.442, *P* < 0.001) experienced significantly lower survival rates within a 30-day follow-up period.

**Conclusion:**

Elevated levels of FPR and CPR serve as distinct risk factors for mortality among patients with SFTS, indicating their potential to predict an unfavorable prognosis in these patients.

## 1 Introduction

Severe fever with thrombocytopenia syndrome (SFTS) is an emerging infectious disease triggered by the SFTS virus (SFTSV), now recognized as *Dabie bandavirus*. In 2011, the Chinese Center for Disease Control and Prevention successfully isolated SFTSV from serum samples obtained from infected patients (Yu et al., 2011). According to the International Committee on Taxonomy of Viruses, SFTSV belongs to the genus *Bandavirus* within the family Phenuiviridae (Adams et al., 2016). Reports of SFTS cases emerged in Korea and Japan in 2012 and 2013, respectively (Kim et al., 2013; Takahashi et al., 2014). Additionally, a related virus called heartland virus was identified in the United States (McMullan et al., 2012). In 2019, retrospective case studies conducted in Vietnam confirmed local transmission of SFTSV (Tran et al., 2019). It has since spread globally, with China reporting 11,995 cases across 25 provinces by 2018 and laboratory-confirmed cases reaching up to 7,721, with a mortality rate as high as 10.5% (Miao et al., 2021). The primary means of transmission for SFTS is via tick bites. However, it has also been demonstrated that direct contact with infected blood or bodily fluids can also lead to human-to-human transmission (Yoo et al., 2018). The clinical presentation of SFTS is non-specific, predominantly marked by fever, fatigue, muscle discomfort, gastrointestinal manifestations, and profound thrombocytopenia. The pathogenesis of SFTS remains elusive and currently there are no vaccines or specific antiviral drugs available for treating SFTSV infection. However, several studies have confirmed the therapeutic benefit of favipiravir in SFTS (Li H. et al., 2021; Yuan et al., 2021). Clinical management primarily involves providing supportive care for symptoms (Liu Y. et al., 2022). In 2017, the World Health Organization (WHO) designated SFTS as an infectious disease requiring urgent research and development efforts due to its high fatality rate and contagiousness (Mehand et al., 2018). The disease can rapidly progress to multiple organ dysfunction syndrome (MODS) resulting in unfavorable clinical outcomes for patients with severe conditions. Therefore timely identification of patients with SFTS with a poor prognosis is crucial.

Several studies have reported a high prevalence of inflammation and malnutrition among patients with SFTS, which often indicates a poor prognosis (Liu Z. et al., 2022). C-reactive protein (CRP), an acute phase protein synthesized by the liver in response to infection or inflammation, exhibits rapid elevation shortly after onset and serves as a sensitive marker for tissue damage and inflammation (Yang et al., 2022). Fibrinogen (FIB), another acute phase reaction protein produced by the liver, is associated with inflammatory processes. Prealbumin (PA) serves as both a nutritional biomarker, commonly used to assess the nutritional status of patients, and as a negative acute phase protein (Sneh et al., 2020). Composite biomarkers such as the CRP to PA ratio (CPR) and the FIB to PA ratio (FPR) concurrently depict both inflammatory and nutritional status. However, prior studies have not investigated whether CPR and FPR could predict the prognosis of patients with SFTS. Therefore, this study seeks to determine whether CPR and FPR could serve as early indicators for identifying poor prognosis in patients with SFTS, offering valuable insights for clinical decision making and treatment strategies.

## 2 Methods

### 2.1 Patients

This retrospective study enrolled 159 hospitalized patients diagnosed with SFTS at the First Affiliated Hospital of Anhui Medical University between September 2019 and June 2023. Diagnosis was confirmed by testing for SFTSV RNA or SFTSV IgM/IgG in blood samples collected upon admission. Among these patients, 116 were diagnosed using SFTSV RNA, while 43 were diagnosed using SFTSV IgM/IgG. Patients were then categorized into two groups based on clinical outcomes: the death group consisting of 28 patients and survival group comprising 131 patients. The study received approval from the research ethics committee of the First Affiliated Hospital of Anhui Medical University (No. PJ20210618) and adhered to the principles outlined in the Declaration of Helsinki. Patient information was anonymized, rendered non-identifiable, and handled confidentially. Patients and their families were informed about the study, and they provided informed consent by signing relevant consent forms.

### 2.2 Data collection

Data were collected from the electronic medical records, encompassing demographic details, comorbidities, clinical presentations, outcomes, and laboratory findings. The laboratory employed in this study holds ISO 15189 certification, with coefficients of variation for both inter- and intra-batch analyses remaining below 8%. The laboratory assessments conducted within 24 hours of admission include routine blood tests using the XN-9000 automatic hematology analyzer (Sysmex, Japan), coagulation function tests with the STA-R Max automatic coagulometer (Stago, France), biochemical marker and C-reactive protein (CRP) analysis utilizing the VITROS-5600 automatic biochemical analysis system (Ortho, USA), and procalcitonin (PCT) tests via the mini VIDAS (Biomerieux, France). Mortality at 30 days constituted the primary endpoint. Furthermore, patients who were discharged within this timeframe were followed up by telephone to determine their outcomes. Mortality, in this context, denotes patients who passed away at any point during the progression of the illness.

### 2.3 Statistical analysis

Continuous variables are presented as either mean ± standard deviation (SD) for normally distributed data or median (M) with interquartile range (IQR) for skewed distributions, while categorical variables are described using frequencies. The student *t*-test or Mann-Whitney U test was utilized to compare differences in value between the two groups. The chi-squared test was employed to determine the difference between categorical variables among groups. Univariate and multivariate logistic regression analysis were conducted to identify independent risk factors for mortality. Variables with a *P-*value of less than 0.1 in the univariate logistic regression analysis were included in the multivariate logistic analysis using a forward logistic regression (LR) method. Additionally, covariates such as age and sex were incorporated as adjusted variables. Receiver operating characteristic (ROC) curve analysis was performed to calculate the optimal cut-off value for risk factors. The area under ROC curve (AUC) with the highest Youden’s index was used to evaluate the predictive efficacy of the risk factors. Survival analysis was conducted using the Kaplan–Meier curve based on the log-rank test. Statistical analyses were carried out using SPSS 21.0 software (IBM, Armonk, NY, USA) and GraphPad Prism 9 software (GraphPad software, San Diego, CA, USA). A *P*-value of < 0.05 was considered as a statistically significant difference.

## 3 Results

### 3.1 Demographics and clinical characteristics of patients with SFTS

**Figure 1:**
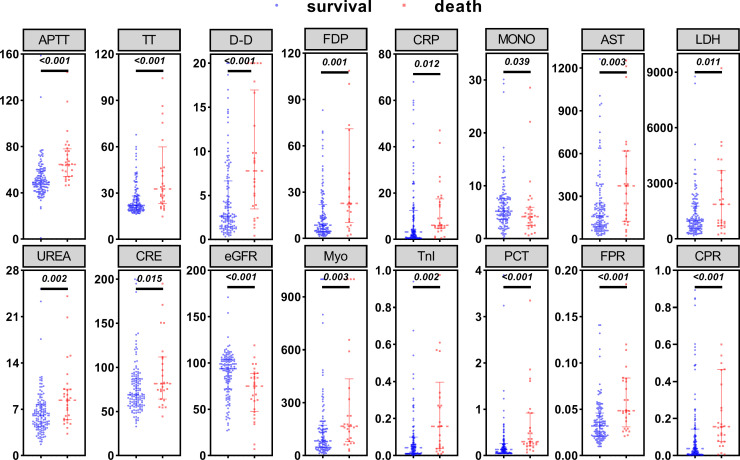
Comparison of laboratory parameters between the survival and death groups. Statistical significance was calculated by the Mann-Whitney U test. Data are presented as median (IQR). APTT, activated partial thromboplastin time; TT, thrombin time; D-D, d-dimer; FDP, Fibrinogen degradation products; MONO, monocyte; AST, aspartate aminotransferase; LDH, lactate dehydrogenase; UREA, urea nitrogen; CRE, creatinine; eGFR, estimated glomerular filtration rate; Myo, myoglobin; TnI, cardiac troponin I; PCT, procalcitonin; FPR, fibrinogen/prealbumin; CPR, C-reactive protein/prealbumin.

A total of 159 patients diagnosed with SFTS were included in the study, with an average age of 63.98 ± 11.25 years. Among these, 71(44.65%) were male and 88(55.35%) were female. Throughout the period of hospitalization, 28 patients experienced fatal outcomes, with MODS being the leading cause of death. The overall 30-day mortality rate was 17.6% (28/159). Fever was the initial symptom observed in all patients (159/159), with the highest body temperature recorded in patients being 38.9°C (38.4°C, 39.0°C). The clinical characteristics and laboratory indicators of patients in both the death group and survival group are summarized in **Tables 1**, **2**, and **Figure 1**.

**Table 1:** Comparison of clinical characteristics between the survival and death groups.

	Survival (n=131)	Death (n=28)	*p* value
Demographic information			
Gender, male (%)	60 (45.8)	11 (39.3)	0.529
Age (years)	63.2 ± 11.5	66.5 ± 9.6	0.530
Hospital stay (days)	11.0 (9.0, 16.0)	5.5 (2.3, 8.0)	<0.001
Onset to admission (days)	5 (4, 7)	5 (4, 7)	0.275
Clinical manifestations			
Highest body temperature (°C)	38.9 (38.4, 39.0)	39.0 (38.4, 39.6)	0.219
Fever, N (%)	131 (100)	28 (100)	–
Vomit, N (%)	35 (26.7)	17 (60.7)	0.001
Headache, N (%)	40 (30.5)	15 (53.6)	0.002
Conscious Disturbance, N (%)	0	26 (92.9)	<0.001
Diarrhea, N (%)	35 (26.7)	11 (39.3)	0.183
Complications			
Hemorrhage of digestive tract, N (%)	20 (15.3)	11 (39.3)	0.004
Pulmonary infection, N (%)	22 (16.8)	12 (42.9)	0.002
Acute pancreatitis, N (%)	31 (23.7)	11 (39.3)	0.089
MODS, N (%)	0	17 (60.7)	<0.001
DIC, N (%)	0	2 (7.1)	0.002

Data are presented as median (IQR), mean ± SD, or N (%). *P*-values were calculated using the Mann-Whitney U test, student t test, or Chi-square test. MODS, multiple organ dysfunction syndrome; DIC, diffuse intravascular coagulation.

**Table 2:** Comparison of laboratory indicators of patients between the survival and death groups.

	Normal range	Survival (n=131)	Death (n=28)	*p* value
Coagulation function				
PT (s)	11.00–16.00	13.10(12.60, 13.70)	13.50(12.80, 14.20)	0.145
APTT (s)	28.00–42.00	49.10(43.10, 58.60)	64.50(54.20, 78.10)	<0.001
FIB (g/L)	2.00–4.00	2.68(2.25, 3.19)	2.41(2.04, 2.98)	0.104
TT (s)	14.00–21.00	22.00(19.40, 28.20)	32.65(23.18, 59.88)	<0.001
D-D (mg/ml)	0.00–0.50	2.64(1.44, 6.65)	7.79(3.48, 16.97)	<0.001
FDP (mg/ml)	0.00–5.00	8.62(4.55, 21.86)	22.76(10.38, 71.08)	0.001
Blood routine				
White blood cell (x10^9^/L)	3.50–9.50	2.43(1.54, 4.50)	2.69(1.44, 3.54)	0.496
Neutrophil (x10^9^/L)	1.80–6.30	1.47(0.94, 2.73)	1.69(0.85, 2.58)	0.760
Lymphocyte (x10^9^/L)	1.10–3.20	0.65(0.41, 1.10)	0.67(0.39, 0.84)	0.359
MONO (x10^9^/L)	0.10–0.60	0.12(0.07, 0.27)	0.08(0.06, 0.14)	0.039
Hemoglobin (g/L)	130.00–175.00	129.00 ± 18.00	126.00 ± 24.00	0.245
Platelet count (x10^9^/L)	125.00–350.00	48.00(34.00, 67.00)	38.00(28.00, 51.00)	0.075
Biochemical markers				
Total protein (g/L)	63.00–82.00	63.69 ± 7.34	63.11 ± 7.85	0.836
PA (mg/L)	180.00–390.00	97.19 ± 47.85	51.39 ± 33.17	0.098
ALT (U/L)	0–35.00	67.00(40.00, 103.00)	99.00(45.00, 211.00)	0.154
AST (U/L)	14.00–36.00	163.00(86.00, 257.00)	374.00(125.00, 619.00)	0.003
LDH (U/L)	120.00–246.00	956.00(555.00, 1770.00)	1986.00(740.00, 4133.00)	0.011
UREA (mmol/L)	2.50–6.10	6.06(4.40, 7.70)	8.36(5.45, 10.05)	0.002
CRE (umol/L)	46.00–92.00	69.00(57.40, 87.30)	82.00(66.30, 116.60)	0.015
eGFR (ml/min)	>90.00	94.00(72.00, 104.00)	75.00(48.00, 89.00)	<0.001
Myo (ng/ml)	10.00–46.00	83.50(44.75, 172.25)	166.50(79.25, 435.00)	0.003
TnI (ng/ml)	0–0.019	0.050(0.011, 0.100)	0.158(0.039, 0.396)	0.002
Lipase (U/L)	23.00–300.00	580.00(313.50, 889.00)	541.00(349.30, 918.50)	0.773
Infection-related biomarkers				
CRP (mg/L)	0.00–10.00	3.20(0.93, 12.49)	6.05(4.78, 17.50)	0.012
PCT (ng/ml)	0.00–0.50	0.13(0.06, 0.26)	0.30(0.21, 0.92)	<0.001
Combined biomarkers				
FPR		0.032(0.021, 0.046)	0.049(0.031, 0.084)	<0.001
CPR		0.038(0.011, 0.143)	0.156(0.076, 0.465)	<0.001

Data are presented as median (IQR). *P*-values were calculated using the Mann-Whitney U test. PT, prothrombin time; APTT, activated partial thromboplastin time; FIB, fibrinogen; TT, thrombin time; D-D, d-dimer; FDP, Fibrinogen degradation products; MONO, monocyte; PA, prealbumin; ALT, alanine aminotransferase; AST, aspartate aminotransferase; LDH, lactate dehydrogenase; UREA, urea nitrogen; CRE, creatinine; eGFR, estimated glomerular filtration rate; Myo, myoglobin; TnI, cardiac troponin I; CRP, C-reactive protein; PCT, procalcitonin; FPR, FIB/PA; CPR, CRP/PA.

There was no significant difference in sex and age between the groups of patients who survived and those who did not (*P* > 0.05), while patients in the death group had shorter hospital stays compared to those in the survival group (*P* < 0.05). In terms of clinical symptoms, the group that experienced mortality exhibited higher frequencies of vomiting, headaches, and disturbances in consciousness. Additionally, there was significantly higher occurrence of MODS, diffuse intravascular coagulation (DIC), gastrointestinal bleeding, and pulmonary infections in the death group compared to the survival group.

The death group exhibited significantly elevated levels of activated partial thromboplastin time (APTT), thrombin time (TT), d-dimer (D-D), fibrinogen degradation products (FDP), C-reactive protein (CRP), aspartate aminotransferase (AST), lactate dehydrogenase (LDH), urea nitrogen (UREA), creatinine (CRE), myoglobin (Myo), cardiac troponin I (TnI), procalcitonin (PCT), fibrinogen-to-albumin ratio (FPR), and C-reactive protein ratio (CPR) compared to the survival group (*P* < 0.05). Conversely, the death group showed significantly lower levels of monocyte (MONO) and estimated glomerular filtration rate (eGFR) compared to the survival group (*P* < 0.05). No statistically significant differences were observed between the two groups for the remaining biomarkers (*P* > 0.05).

### 3.2 Independent risk factors for mortality in patients with SFTS

We identified independent risk factors for patients with SFTS to enable early identification of severe cases and predict clinical outcomes effectively. In our analysis differential clinical indicators were included in the regression analysis. As presented in **Table 3**, univariate logistic regression analysis revealed that elevated APTT (OR = 5.987, 95% CI: 2.427 - 14.771, *P* < 0.001), TT (OR = 4.162, 95% CI: 1.774 - 9.763, *P* = 0.001), D-D (OR = 4.044, 95% CI: 1.679 - 9.744, *P* = 0.002), FDP (OR = 3.538, 95% CI: 1.480 - 8.456, *p* = 0.004), AST (OR = 4.032, 95% CI: 1.707 - 9.523, *P* = 0.001), LDH (OR = 3.258, 95% CI: 1.407 - 7.542, *P* = 0.006), UREA (OR = 3.440, 95% CI: 1.466 - 8.072, *P* = 0.005), TnI (OR = 4.242, 95% CI: 1.501 - 11.990, *P* = 0.006), PCT (OR = 4.049, 95% CI: 1.62 - 10.118, *P* = 0.003), FPR (OR = 6.141, 95% CI: 2.531 - 14.899, *P* < 0.001) and CPR (OR = 10.863, 95% CI: 3.121 - 37.808, *P* < 0.001), as well as decreased PA (OR = 6.476, 95% CI: 2.129 - 19.702, *P* = 0.001), MONO (OR = 0.341, 95% CI: 0.111 - 1.045, *P* = 0.060) and eGFR (OR = 4.035, 95% CI: 1.688 - 9.642, *P* = 0.002), were associated with a higher risk of poor prognosis.

**Table 3:** Logistic regression analysis of risk factors for prognosis in patients with SFTS.

Variable	Classification	Univariate analysis		Multivariate analysis^a^		Multivariate analysis^b^	
		OR (95%CI)	*p*	OR (95%CI)	*p*	OR (95%CI)	*p*
Age (years)	≥65 vs. <65	2.017 (0.850–4.784)	0.112				
Sex	Male vs. Female	0.766 (0.333–1.761)	0.530				
PA (mg/L)	<90 vs. ≥90	6.476 (2.129–19.702)	0.001				
FIB (g/L)	<2.8 vs. ≥2.8	1.738 (0.741–4.074)	0.204				
APTT (s)	≥55 vs. <55	5.987 (2.427–14.771)	<0.001				
TT (s)	≥30 vs. <30	4.162 (1.774–9.763)	0.001				
D-D (mg/ml)	≥5.5 vs. <5.5	4.044 (1.679–9.744)	0.002	4.291 (1.415–13.016)	0.010	4.291 (1.415–13.016)	0.010
FDP (mg/ml)	≥22 vs. <22	3.538 (1.480–8.456)	0.004				
CRP (mg/L)	≥10 vs. <10	1.381 (0.583–3.271)	0.463				
MONO (x10^9^/L)	≥0.2 vs. <0.2	0.341 (0.111–1.045)	0.060				
AST (U/L)	≥275 vs. <275	4.032 (1.707–9.523)	0.001				
LDH (U/L)	≥1700 vs. <1700	3.258 (1.407–7.542)	0.006				
UREA (mmol/L)	≥6.9 vs. <6.9	3.440 (1.466–8.072)	0.005	3.726 (1.23–11.285)	0.020	3.726 (1.23–11.285)	0.020
CRE (umol/L)	≥85 vs. <85	1.769 (0.766–4.086)	0.182				
eGFR (ml/min)	<85 vs. ≥85	4.035 (1.688–9.642)	0.002				
Myo (ng/ml)	≥190 vs. <190	2.014 (0.818–4.955)	0.128				
TnI (ng/ml)	≥0.3 vs. <0.3	4.242 (1.501–11.990)	0.006				
PCT (mg/L)	≥0.47 vs. <0.47	4.049 (1.62–10.118)	0.003				
FPR	≥0.45 vs. <0.45	6.141 (2.531–14.899)	<0.001	6.578 (2.120–20.414)	0.001	6.578 (2.120–20.414)	0.001
CPR	≥0.05 vs. <0.05	10.863 (3.121–37.808)	<0.001	6.715 (1.686–26.747)	0.007	6.715 (1.686–26.747)	0.007

^a^Unadjusted, ^b^adjusted for age and sex. FIB, fibrinogen; PA, prealbumin; APTT, activated partial thromboplastin time; TT, thrombin time; D-D, d-dimer; FDP, Fibrinogen degradation products; MONO, monocyte; AST, aspartate aminotransferase; LDH, lactate dehydrogenase; UREA, urea nitrogen; CRE, creatinine; eGFR, estimated glomerular filtration rate; Myo, myoglobin; TnI, cardiac troponin I; CRP, C-reactive protein; PCT, procalcitonin; FPR, FIB/PA; CPR, CRP/PA; CI, confidence interval; OR, odd ratio.

Variables with a *P*-value less than 0.1 in the univariate logistic regression analysis, including PA, MONO, eGFR, APTT, TT, D-D, FDP, AST, LDH, UREA, TnI, PCT, FPR and CPR, were included in multivariate logistic analysis using a forward LR method. The analysis revealed that elevated levels of D-D (OR = 4.291, 95% CI: 1.415 - 13.016, *P* = 0.010), UREA (OR = 3.726, 95% CI: 1.23 - 11.285, *P* = 0.020), FPR (OR = 6.578, 95% CI: 2.120 - 20.414, *P* = 0.001), and CPR (OR = 6.715, 95% CI: 1.686 - 26.747, *P* = 0.007), independently contributed to a poorer prognosis among patients with SFTS. Notably, there was no change in the odds ratio (OR) values regardless of adjustment for age and sex indicating that FPR and CPR remained stable risk factors for prognosis in patients with SFTS.

### 3.3 Diagnostic efficacy of FPR and CPR on clinical outcomes in patients with SFTS

**Figure 2:**
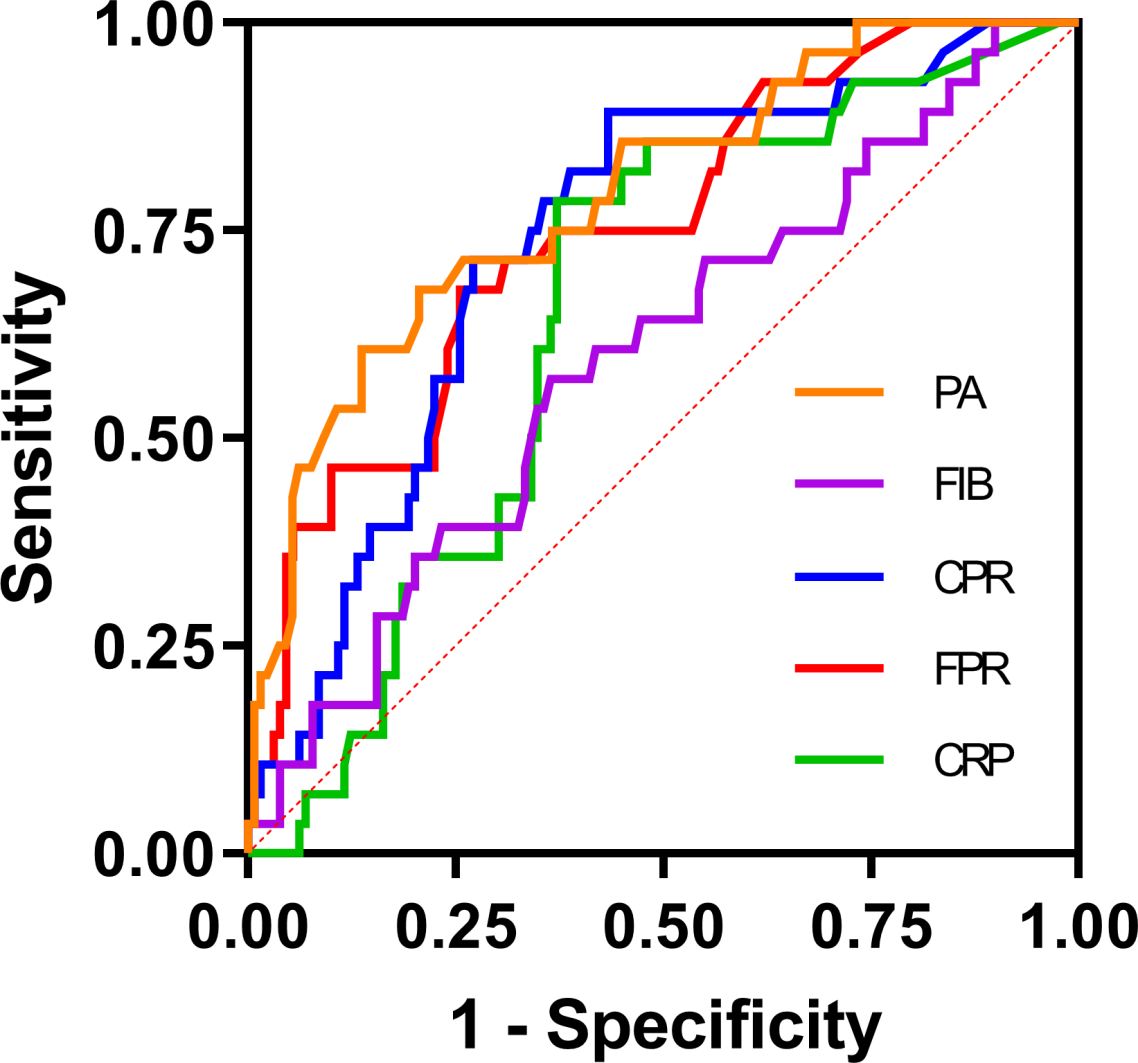
ROC curve analysis of the FPR, CPR, FIB, PA, and CRP for 30-day mortality in patients with SFTS. The ROC curve plots were created with GraphPad Prism 9. The SFTS patients were divided into survival and nonsurvival groups based on their survival status according to the ROC curve analysis. CRP, C-reactive protein; FIB, fibrinogen; PA, prealbumin; FPR, FIB/PA; CPR, CRP/PA.

To evaluate the prognostic significance and determine the optimal cut-off values for FPR, CPR, FIB, PA, and CRP in predicting disease prognosis among patients with SFTS, ROC curve analysis was conducted (**Table 4** and **Figure 2**). The analysis revealed that the AUCs for survival were 0.748 (95% CI: 0.650 - 0.846, *P* < 0.001) for FPR, 0.741 (95% CI: 0.645 - 0.837, *P* < 0.001) for CPR, 0.598 (95% CI: 0.483 - 0.714, *P* = 0.104) for FIB, 0.791 (95% CI: 0.698 - 0.884, *P* < 0.001) for PA and 0.654 (95% CI: 0.554 - 0.754, *P* = 0.011) for CRP, respectively. According to the maximum Youden’s index, the optimal cut-off value for predicting poor prognosis was determined as 0.045 for FPR, with 67.9% sensitivity (95% CI: 47.6% - 83.5%) and 74% specificity (95% CI: 65.4%-81.1%). Similarly, the optimal cut-off value for predicting poor prognosis was 0.05 for CPR, with 89.3% sensitivity (95% CI: 70.6% - 97.2%) and 56.7% specificity (95% CI: 47.7% - 65.3%).

**Table 4:** ROC curve analysis of the FPR, CPR, FIB, PA, and CRP for 30-day mortality in patients with SFTS.

Variable	AUC (95%CI)	*P*	Cut-off value	Sensitivity (95%CI)	Specificity (95%CI)
CRP	0.654 (0.554–0.754)	0.011	4.745	0.786 (0.586–0.910)	0.630 (0.540–0.712)
FIB	0.598 (0.483–0.714)	0.104	2.480	0.571 (0.374–0.749)	0.636 (0.546–0.718)
PA	0.791 (0.698–0.884)	<0.001	48.000	0.607 (0.407–0.779)	0.860 (0.786–0.912)
FPR	0.748 (0.650–0.846)	<0.001	0.045	0.679 (0.476–0.835)	0.740 (0.654–0.811)
CPR	0.741 (0.645–0.837)	<0.001	0.050	0.893 (0.706–0.972)	0.567 (0.477–0.653)

CRP, C-reactive protein; FIB, fibrinogen; PA, prealbumin; FPR, FIB/PA; CPR, CRP/PA.

### 3.4 Survival analysis

**Figure 3:**
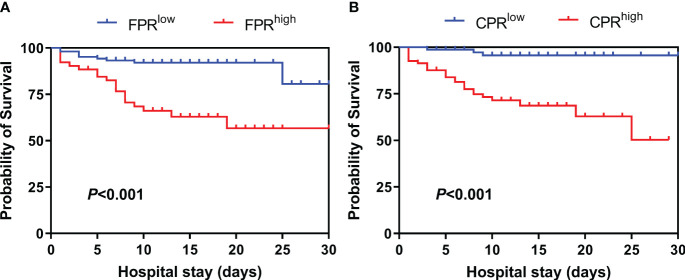
Kaplan–Meier survival curves according to the cut-off value of FPR and CPR. **(A)** FPR, fibrinogen/prealbumin, cut-off = 0.045. **(B)** CPR, C-reactive protein/prealbumin, cut-off = 0.05.

Patients were stratified into two groups, namely FPR^low^ and FPR^high^, utilizing the optimal cut-off value of FPR (cut-off value = 0.045). Similarly, patients were categorized into CPR^low^ and CPR^high^ groups based on the optimal cut-off value of CPR (cut-off value = 0.05). Kaplan–Meier survival analysis demonstrated that patients with FPR > 0.045 (log-rank test; χ2 = 17.370, *P* < 0.001) or CPR > 0.05 (log-rank test; χ2 = 19.442, *P* < 0.001) exhibited significantly lower survival rates during the 30-day follow-up period (**Figure 3**).

## 4 Discussion

As a newly recognized infectious disease, SFTS poses a significant challenge due to its rapid onset and high fatality rate. Early diagnosis and effective management of symptoms and complications are paramount to improving the prognosis of patients with this disease (Liu et al., 2020). Consequently, there is an urgent need for early biomarkers to facilitate in this process. In recent years, FPR and CPR have emerged as promising indicators capable of capturing both systemic inflammation and nutritional status in various diseases. This combined index of the FPR and CPR holds potential as a prognostic biomarker in patients with conditions such as tumors (Matsunaga et al., 2020; Sun et al., 2020; Maruyama et al., 2022; Kwon et al., 2023), infectious diseases (Yue et al., 2015; Bi et al., 2022), autoimmune diseases (Cheng et al., 2021), and other ailments (Zang et al., 2020; Yamada et al., 2021; Huang et al., 2022). To date, no studies have examined whether FPR and CPR can assess the prognosis of patients with SFTS. Therefore, this study appears to be the first to investigate the potential utility of FPR and CPR in predicting the adverse outcomes in prognosis of patients with SFTS.

The observed mortality rate in this study, ranging from 13% to 24% aligns closely with the reported case fatality rates of SFTS documented in existing literature (Miao et al., 2021; Zhao et al., 2022). Notably this study also revealed a higher incidence of clinical symptoms such as vomiting, headache, and consciousness disorder among patients who succumbed to the disease compared to those who survived. This finding is in line with the observations made by Wang et al. (2022), who pointed out that patients presenting with central nervous system symptoms tend to have a higher mortality ratio, consistent with the results of this study.

Furthermore, in our study, we identified MODS as the primary cause of mortality among fatal cases, a trend consistent with the majority of previous research findings (Liu et al., 2020; Miao et al., 2021). In previous studies, age has been widely considered as a significant risk factor for SFTS (Liu et al., 2020). However, there was no significant difference in age distribution between the two groups in our study. One plausible explanation for this discrepancy could be the limited sample size, which may lead to an increased standard error of variables and thus obscure any significant age-related differences.

SFTS can be divided into four distinct phases based on its clinical progression: the latent period (lasting approximately 1 week); the fever period (occurring from days 1–7 after onset); the multiple organ dysfunction period (spanning days 7–13 from onset); and the convalescence phase (Liu et al., 2013). Our study findings revealed that biomarkers indicative of multi-organ injury, such as those related to coagulation function (APTT, TT, D-D, and FDP), infection-related biomarkers (CRP, PCT), hepatocyte damage markers (AST, LDH), renal impairment markers (UREA, CRE), and myocardial injury markers (Myo, TnI), were significantly elevated in patients who died compared to survivors. Additionally, the levels of MONO and eGFR were notably lower in the death group compared to the survival group. These findings suggest that patients in the death group experienced an earlier and more pronounced onset of the multiple organ dysfunction period compared to those in the survival group, a pattern consistent with findings from several prior studies (Ge et al., 2022; Zhao et al., 2022; Liu et al., 2023; Song et al., 2023).

CRP stands as one of the most widely utilized inflammatory markers, typically exhibiting minimal or no increase during viral infections except in cases of severe viral infections that induce tissue and organ damage (Yang et al., 2022). Previous studies have highlighted the efficacy of CRP in predicting mortality in SFTS cases (Liu Z. et al., 2022). However, in our study while CRP levels were higher in the death group compared to the survival group, its predictive capacity for mortality outcomes was limited as evidenced by an AUC of 0.654. The timing of blood sample collection may contribute to this observation. A systematic review suggested that CRP levels obtained early (within 48 hours) may not reliably predict survival in critically ill patients, while late (beyond 48 hours) CRP levels might possess predictive value (Zhang and Ni, 2011).

Compared to other nutritional markers like albumin and total cholesterol, PA can provide a more precise and sensitive assessment of the nutritional status of the patient upon admission primarily due to its relatively short half-life of two days. Additionally, during inflammatory processes, cytokines such as IL-6, IL-1, and TNF-α also contribute to the decrease of PA levels (Yue et al., 2015). The prognostic potential of PA has been demonstrated in various conditions including acute kidney injury (Xie et al., 2011), tumors (Kwon et al., 2023), infectious diseases (Bi et al., 2022), and heart failure (Lourenço et al., 2014). Although PA demonstrated a good predictive ability for mortality among patients with SFTS (AUC = 0.791), statistical significance was not achieved in the univariate analysis (*P* = 0.098), likely due to the limited sample size. Fibrinogen, a hepatically synthesized glycoprotein plays a pivotal role in regulating the hemostatic system, coagulation, and systemic inflammation (Hou et al., 2022).

We examined the combination of three readily available clinical laboratory parameters, CRP, FIB, and PA. Our findings for the first time revealed that the elevated levels of FPR and CPR independently contribute to mortality risk in patients with SFTS, serving as predictors of unfavorable prognosis. Additionally, the results of this study indicate that D-D and UREA are also independent risk factors for fatal outcomes in patients with SFTS, aligning with the conclusions of numerous prior studies (Gui et al., 2021; Wang et al., 2022). This observation may be linked to coagulation dysfunction as evidenced by the rapid increase in D-D levels among certain critically ill patients with SFTS, indicating the presence of a hypercoagulable state and secondary hyperfibrinolysis, ultimately culminating in DIC and posing a significant risk for mortality (Zhang et al., 2012). Furthermore, heightened levels of UREA, a byproduct of protein catabolism, in critically ill patients underscores the intensified protein breakdown observed in such cases (Li X. et al., 2021).

The observed influence remained significant even after controlling for various confounding variables. The ROC curve analysis revealed that FPR (AUC = 0.748) and CPR (AUC = 0.741) exhibited strong discriminatory power in predicting mortality in patients with SFTS, outperforming CRP (AUC = 0.654) and FIB (AUC = 0.598). Moreover, in the survival analysis, patients exhibiting elevated FPR and CPR demonstrated a significantly diminished likelihood of 30-day survival. A prospective study from China demonstrated that CPR was independently associated with in-hospital mortality in the medical intensive care unit (Li et al., 2017). Similarly, another study analyzing 169 patients with acute pancreatitis highlighted FPR as a superior prognostic indicator compared to CRP in predicting the prognosis of these patients (Yue et al., 2015). The correlation between elevated FPR and CPR and poor prognosis in patients with SFTS can be elucidated by the comprehensive assessment of both inflammatory and nutritional status, crucial for predicting outcomes in patients with SFTS. Despite the potential for mutual influence between these factors, they exhibit a robust correlation with clinical outcomes. Additionally, alterations in either marker can affect this ratio, potentially enhancing sensitivity compared to individual markers alone.

Furthermore, CRP, FIB, and PA not only serve as indicators but also possess distinct pathophysiological functions implying their involvement in disease progression and association with unfavorable prognosis. However, further investigations are required to comprehensively elucidate the underlying mechanisms. Consequently, healthcare professionals should diligently monitor patients with SFTS with elevated FPR and CPR levels and promptly implement appropriate management strategies to enhance prognosis.

However, this study also possesses several limitations. Firstly, it was a retrospective, single-center study with a relatively small cohort size and the number of deaths was limited, potentially introducing statistical bias. Secondly, we only assessed FPR and CPR at admission, neglecting the potential value of continuous monitoring to evaluate disease progression over time. These limitations underscore the need for further investigations in future studies.

## 5 Conclusion

The association between inflammation and malnutrition is complex and interdependent, with inflammation potentially contributing to malnutrition, while malnutrition can also influence the development of inflammation (Massironi et al., 2023). The findings of this study suggest that FPR and CPR, when employed as combined biomarkers reflecting both systemic inflammation and nutritional status, emerge as independent risk factors with substantial predictive value for poor prognosis among patients with SFTS. These indicators are easily accessible, cost-effective, and reproducible, facilitating prompt identification of high-risk patients, early intervention, and dynamic monitoring of their condition.

## Data availability statement

The raw data supporting the conclusions of this article will be made available by the authors, without undue reservation.

## Ethics statement

This study was approved by the research ethics committee of the First Affiliated Hospital of Anhui Medical University (No. PJ20210618) and performed in accordance with the principles of the Declaration of Helsinki. Patient information was anonymized, non-identifiable, and treated confidentially. Patients and their families were informed about this study and signed the relevant informed consent forms. The studies were conducted in accordance with the local legislation and institutional requirements. The participants provided their written informed consent to participate in this study.

## Author contributions

FZ: Conceptualization, Data curation, Formal analysis, Writing – original draft, Writing – review & editing. XL: Conceptualization, Data curation, Formal analysis, Writing – original draft, Writing – review & editing. JQ: Conceptualization, Data curation, Formal analysis, Writing – original draft, Writing – review & editing. WH: Conceptualization, Data curation, Formal analysis, Writing – original draft, Writing – review & editing.
